# Breath-holding could improve visualization of the internal jugular veins by ultrasound guidance in obese patients with trauma

**DOI:** 10.1186/s13049-022-01007-3

**Published:** 2022-03-15

**Authors:** Qi Chen, Jing-qiu Liang, Ran An, Hong-liang Liu

**Affiliations:** 1 Department of Anesthesiology, Chongqing University Cancer Hospital, Chongqing, China; 2 Chongqing Cancer Multi-Omics Big Data Application Engineering Research Center, Chongqing University Cancer Hospital, Chongqing, China

To the Editor,

At present, the internal jugular vein is still the first choice for deep vein cannulation. Although it has a higher incidence of deep vein thrombosis than the subclavian vein, it has fewer complications and is easy to perform [1]. Furthermore, the internal jugular vein is relatively superficial, making it very convenient to use ultrasound guidance for its visualization. However, clinical observations have shown that the cross-sectional area (CSA) of the internal jugular vein varies greatly. Studies have reported that the CSA is related to age, sex, and cervical location [2]. Fasting, obesity, trauma and other factors can also cause certain difficulties in internal jugular vein cannulation. Ultrasound is currently an effective visualization method that can increase the success rate of cannulation compared to anatomic landmark technique. However, the internal jugular vein itself is not filled, which causes difficulty in cannulation and catheterization.

**Fig. 1:**
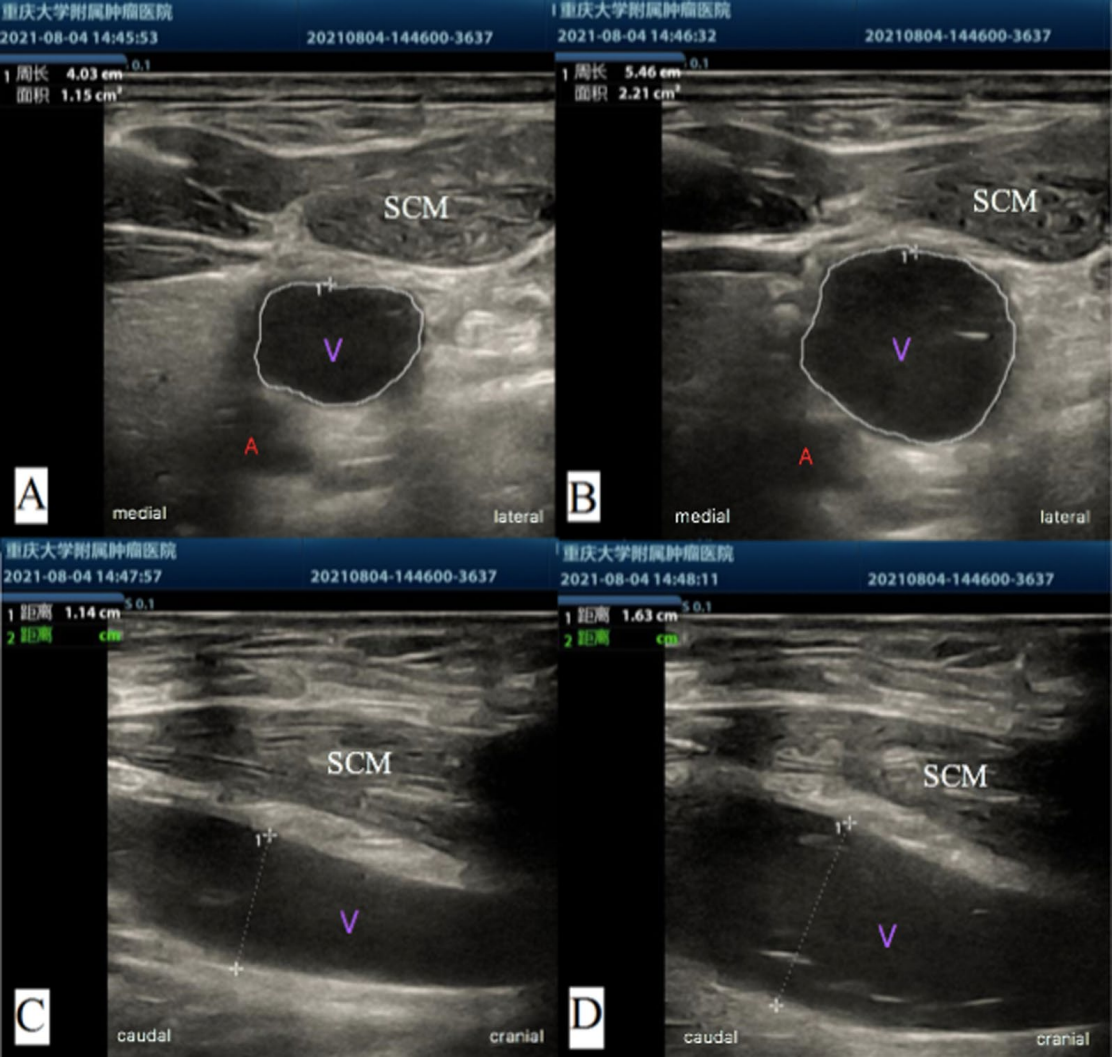
Different filling states of the internal jugular vein. V: internal jugular vein; A: Carotid artery; SCM: Sternocleidomastoid muscle. **A** Short axis of internal jugular vein on clam breath; **B** short axis of internal jugular vein on breath-holding; **C** long axis of the internal jugular vein on clam breath; **D** long axis of the internal jugular vein on breath-holding

In clinical practice, commonly used methods to increase the filling of the internal jugular vein include the Trendelenburg position, elevation of the patient's lower limbs, and the Valsalva maneuver. Studies have shown that the 10-degree Trendelenburg position can increase the CSA of the right and left internal jugular veins by 46% and 41%, respectively, whereas the 45-degree passive leg raise can only increase it by 14% and 8%, respectively [3]. In contrast, some studies have proposed that lowering the head to a 15-degree Trendelenburg position increased the mean CSA by only 17%, which was not statistically significant [4]. For obese patients, the effect of the Trendelenburg position is even more controversial. Studies have pointed out that the Trendelenburg position does not increase the CSA of the internal jugular vein in obese patients [5]. Therefore, it is particularly important to identify whether there is a more convenient and effective method to improve the CSA of the internal jugular vein and increase the success rate of catheterization. Although studies have shown that breath-holding can increase the CSA of the right internal jugular vein by 55%, only healthy volunteers were included [3]. Regarding patients undergoing surgery, most of them are in a state of lack of capacity with poor filling of the internal jugular vein. It is unclear whether the same effect could be achieved (Fig. 1).

We selected 10 fracture traumatic patients who required internal jugular vein catheterization and had a body mass index greater than 30. During the cannulation process, real-time ultrasound positioning combined with the breath-holding method was used. The operation process and data publications were approved by the patients. We performed routine disinfection and preparation and followed strict aseptic requirements to prepare high-frequency ultrasound. The ultrasound probe was placed at the right C5 level, and the carotid artery and internal jugular vein were placed in the center of the field of view; the cross-sectional image of the internal jugular vein was taken after the patient breathed calmly, and held the breath after inhaled deeply. The ultrasound probe was rotated to the long axis of the internal jugular vein, and the image was captured during the patient’s calm breath and deep breath hold. After breath-holding, cannulation in the long-axis plane under real-time ultrasound guidance was performed. After inserting the guide wire, the patient was instructed to resume breathing. In all 10 patients, the success rate of first needle cannulation and catheterization was 100%. After the procedures, the CSA of the internal jugular vein and the diameter under the long axis were measured at two time points. The internal jugular veins were not well filled, and the volume was relatively insufficient when the ten patients breathed calmly. After breath-holding, the cross-sectional area of the internal jugular vein expanded by an average of 72%, while the diameter under the long axis increased by an average of 32%. These changes should be related to the increase in intrathoracic pressure during breath-holding and the obstruction of internal jugular vein return. There are also many clinical Valsalva movements that can achieve similar results, but the patient coordination is often poor, and breath-holding is relatively simple and easy for the patient.

Based on our findings, we concluded that breath-hold could be a beneficial and safe procedure to provide better internal jugular vein puncture conditions in traumatic obese patients, which will help improve the success rate of catheterization.
